# Early middle meningeal artery embolization for small acute epidural hematomas: a clinical study from a single center

**DOI:** 10.3389/fneur.2026.1760515

**Published:** 2026-02-18

**Authors:** Wei Wang, Xiaosheng Yang, Zhaoliang Sun, Weijie Zhong, Yingfan Xiong, Yang Wang, Zhixin Duan, Shenghua Chu, Xiufeng Jiang, Yi Li

**Affiliations:** Department of Neurosurgery, Shanghai Jiao Tong University School of Medicine Affiliated Ninth People's Hospital, Shanghai, China

**Keywords:** embolization, endovascular treatment, epidural haematoma, middle meningeal artery (MMA), skull fracture

## Abstract

**Objective:**

Small acute epidural hematomas (EDHs) are generally managed conservatively; however, in certain instances, they may necessitate surgical evacuation owing to delayed hematoma expansion. This study aims to evaluate whether early embolization of the middle meningeal artery (MMA) can prevent hematoma progression in patients with small acute epidural hematomas accompanied by skull fractures involving meningovascular structures. We hypothesize that early MMA embolization could be a prophylactic strategy to prevent hematoma progression.

**Methods:**

A retrospective study was conducted on 14 patients with small EDHs (volume <30 mL) and skull fractures intersecting MMA branches, who were treated via endovascular embolization at our center from October 2021 to August 2024. Onyx^™^-18 was utilized for embolization after vascular injury detection via super-selective digital subtraction angiography (DSA). The study meticulously documented and analyzed clinical data, imaging findings, surgical outcomes, and prognosis.

**Results:**

Preoperative computerized tomography (CT) scans confirmed skull fractures at EDH sites, and preoperative angiography showed vascular lesions of the MMA (including active contrast leak or pseudoaneurysm) in all cases. All patients underwent the procedure under local anesthesia and obtained an MMA occlusion successfully, which was confirmed by subsequent angiography. All patients showed no hematoma expansion on follow-up CT scans and avoided craniotomy. All patients were discharged without neurological deficits.

**Conclusion:**

This series establishes MMA embolization as a prophylactic approach for small acute EDHs with skull fractures intersecting MMA branches. Early super-selective angiography is crucial for detecting vascular injuries, and subsequent embolization guided by intraoperative findings may reduce monitoring burden and the risk of delayed expansion in selected patients.

## 1 Introduction

Traumatic epidural hematoma (EDH) is a devastating neurosurgical condition due to traumatic brain injury and is commonly associated with injury to the MMA caused by skull fractures (1). Large rapidly progressive EDHs are classically attributed to laceration of the MMA near the foramen spinosum, whereas smaller EDHs may arise from distal MMA branch injury and can be associated with diploic venous injury along the fracture line (2). Typically, surgical evacuation is recommended when hematoma volume exceeds 30 mL, the maximum thickness exceeds 15 mm, the midline shift exceeds 5 mm, and the Glasgow Coma Scale score is below 8 (3, 4). Instead, patients with small EDHs do not meet the surgical indications, which are usually treated conservatively with close observation and repeated CT scans dynamically because the small EDHs may unpredictably and suddenly expand, leading to severe neurological deficits (5). The MMA, a terminal branch of the external carotid artery (ECA), courses rigidly within the cranial dura, rendering it vulnerable to fracture-related injuries (6). The MMA serves as a route for endovascular embolization for conditions such as dural arteriovenous fistula (DAVF) and chronic subdural hematoma (CSDH) (7, 8). Emerging evidence indicates that MMA embolization may be a promising treatment option for small EDHs (2, 9, 10). In this article, we describe our experience with the embolization of the MMA to treat 14 small EDH patients and discuss their clinical characteristics, aiming to describe our institutional experience and explore the feasibility of early prophylactic MMA embolization in a fracture-defined high-risk subgroup.

## 2 Methods

### 2.1 Patient population

We retrospectively analyzed data from 14 patients with small acute EDHs who underwent MMA embolization at Shanghai Ninth People’s Hospital, Shanghai Jiao Tong University School of Medicine, between October 2021 and August 2024. All patients underwent a consistent endovascular workflow as described below, based on our institutional practice. During the study period, all consecutive eligible patients underwent super-selective MMA angiography; vascular lesions were identified in all included cases. We recorded patients’ demographic information, clinical manifestations, CT characteristics, angiographic findings, surgical outcomes, and prognosis.

Exclusion criteria included hematoma volume >30 mL, thickness >15 mm, midline shift >5 mm, or the presence of depressed skull fractures. Patients without complete clinical and imaging data were also excluded. In addition, small EDHs without skull fractures were managed conservatively without angiography at our center and therefore were not included in this series.

### 2.2 Standard procedures

Upon arrival at the emergency department, each patient underwent a head CT scan to diagnose the EDH. For those who met the surgical indications, craniotomy was performed immediately. In patients with EDH associated with skull fractures who were not candidates for immediate surgery, super-selective MMA DSA was performed to identify potential vascular lesions. MMA embolization was subsequently performed under local anesthesia. Subcutaneous infiltration of 2% lidocaine was administered at the femoral access site. In addition, prior to dimethylsulfoxide (DMSO) and Onyx^™^-18 injection, intra-arterial lidocaine (2%, ≤50 mg) was delivered through the microcatheter into the MMA to alleviate DMSO-related pain. A 6-French sheath was introduced using the modified Seldinger’s technique. Then, a 6-F Envoy^®^ guiding catheter (Cordis, Miami Lakes, FL, United States) was positioned in the proximal ECA for angiography. The DMSO-compatible Echelon 10 microcatheter (Medtronic, Minneapolis, MN, United States) was cautiously advanced over a Synchro-14 microwire (Stryker, Kalamazoo, MI, United States) selectively into the trunk of the MMA under roadmap guidance for a second angiography to confirm whether there was a laceration of the MMA and assess the potentially dangerous anastomoses. If the anastomoses were identified, the distal end of the microcatheter was placed at the distal side of the anastomosis to avoid accidental embolization. Before DMSO injection, 2% lidocaine (up to 50 mg) was administered through the microcatheter to alleviate DMSO-related pain. Then the OnyxTM-18 (Micro Therapeutics Inc., Irvine, California, United States) was injected with controlled reflux until adequate embolization was achieved. After the withdrawal of the microcatheter, angiography via the guiding catheter confirmed the full resolution of the vascular lesion.

### 2.3 Follow-up

EDHs in all patients were initially diagnosed via CT scan upon admission, with follow-up scans conducted 1 to 7 days post-MMA embolization or earlier if clinically indicated. Radiographic success was defined as no increase in EDH volume; reduction in size and/or density change was considered regression. Clinical and radiographic observation continued through hospitalization; post-embolization care consisted of standard neurological observation, and antifibrinolytic agents, such as tranexamic acid, were not routinely administered following embolization at our center.

## 3 Results

Fourteen patients, consisting of eleven males and three females, were included in the study. The median age of the participants was 40 years, with an age range of 21 to 58 years. The causes of head injury were car accidents (57.1%) and falls (42.9%). Eleven out of the 14 patients (78.6%) presented with a GCS higher than 13 on admission. All of the 14 patients performed CT in the emergency department. Hematomas were located in the temporal area in 9 cases, parietal area in 2 cases, parietal–temporal area in 2 cases, and frontotemporoparietal area in 1 case. The hematoma volume ranged from 2 to 25 mL (mean 11.2 ± 7.3 mL). Skull fractures were present on CT scans in all patients, and other associated injury lesions were listed in Table 1. Clinical manifestations documented in the medical records included headache, dizziness, nausea, and transient loss of consciousness. Symptoms did not worsen during hospitalization, and no patient experienced clinical deterioration requiring craniotomy; all were discharged without neurological deficits. Angiography showed vascular injuries in all cases, corresponding to bleeding from branches of the MMA. Embolization of the MMA using OnyxTM-18 resulted in angiographic confirmation of cessation of extravasation from the injured MMA branch in all cases. Subsequent CT scans (1–7 days post-embolization) indicated no further hematoma expansion or hematoma regression.

**Table 1:** Summary of clinical data in 14 patients with EDH.

GCS score on admission	Number of patients (n)
13–15	11
9–12	3
Trauma mechanism	
Fall	6
car accident	8
EDH topography	
Temporal	9
Parietal	2
Frontotemporoparietal	1
Parietaltemporal	2
EDH laterality	
right	7
left	7
Angiographic finding	
Active contrast extravasation	11
Acute contrast extravasation & pseudoaneurysm	2
Pseudoaneurysm	1
Associated lesions	
Fracture	14
Contusion	11
SAH	9
Pneumocephalus	8

## 4 Representative cases

### 4.1 Case 1

**Figure 1:**
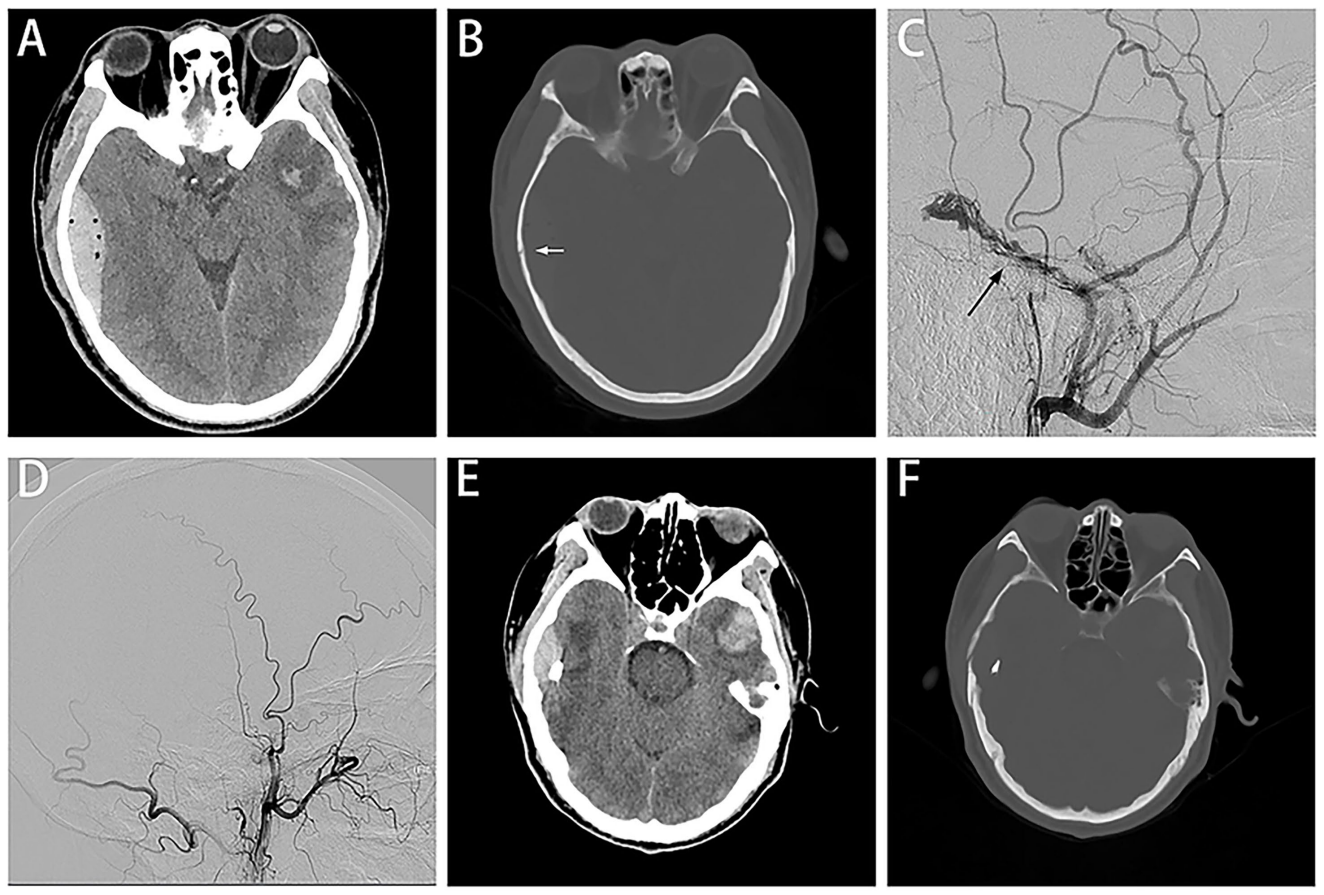
Case 1 representative images. CT scans show right temporal EDH with related fracture (**A,B**; white arrow), selective ECA angiography revealed MMA abnormally expanded and early venous drainage raising suspicion for traumatic DAVF **(C)**, angiographic view after embolization **(D)**, follow-up CT scan demonstrated no evidence of hematoma enlargement **(E,F)**.

A patient in their 50s was injured in a traffic accident and was hospitalized with a headache and dizziness. The GCS score was 14 out of 15. There were no other positive signs except for bleeding from the right external auditory canal. The CT scan revealed a right temporal fracture, a small EDH on the right temporal region, a subarachnoid hemorrhage, pneumocephalus, and a left temporal contusion (Figures 1A,B). Selective right ECA angiography revealed abnormally expanded and noticeable MMA active contrast extravasation with early venous drainage raising suspicion for traumatic DAVF (Figure 1C). To prevent hematoma enlargement, the MMA was embolized using Onyx^™^-18 (Figure 1D). The time from injury to embolization was less than eight hours. The CT scan before discharge showed no hematoma expansion or recurrence (Figures 1E,F).

### 4.2 Case 2

**Figure 2:**
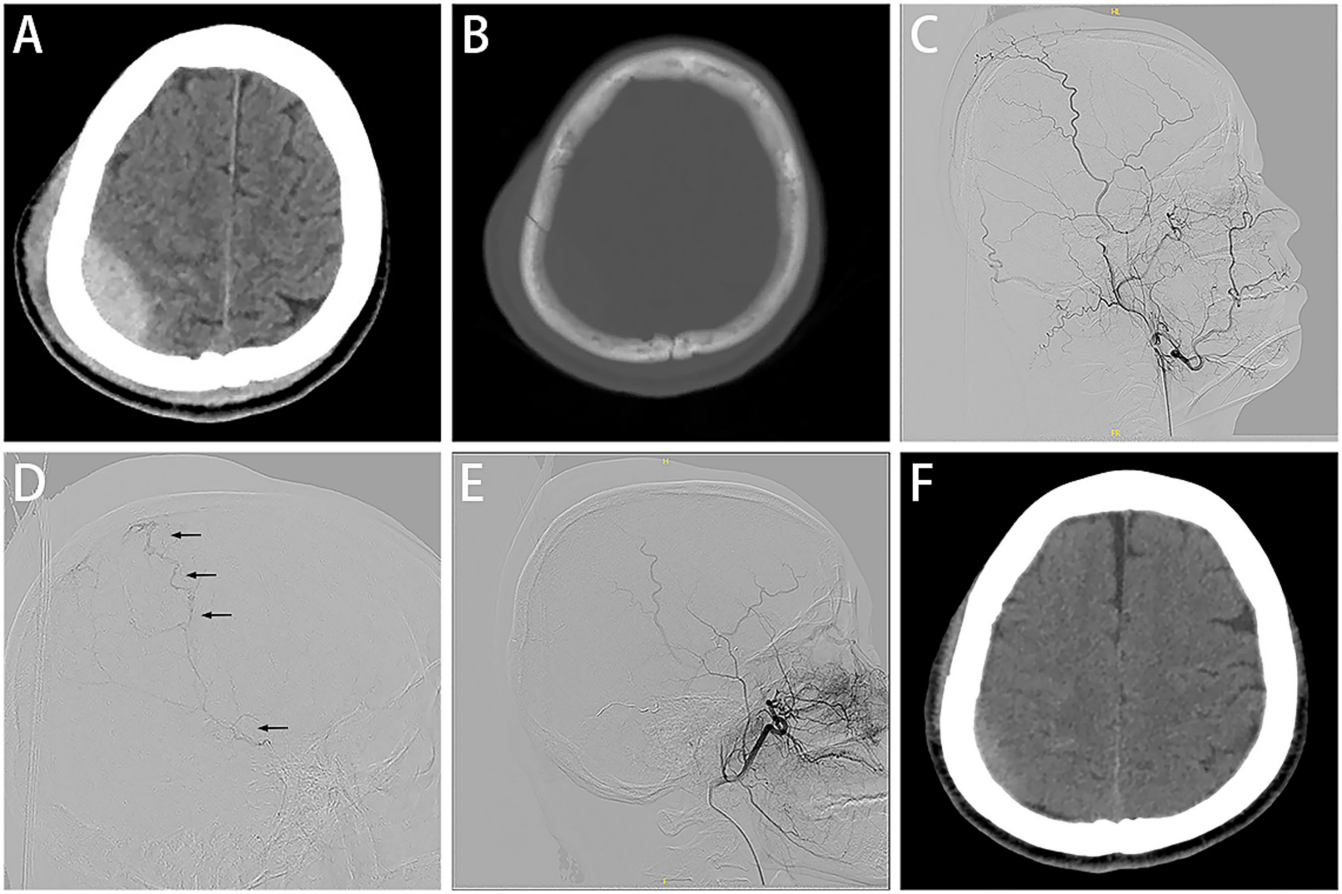
Case 2 representative images. Initial CT scans revealed acute EDH in the right parietal region adjacent to fracture **(A,B)**. Ipsilateral ECA angiography shows no abnormal findings **(C)**. Super-selective MMA angiography via a microcatheter **(D)** shows abnormal dilation of branch vessels and extravasation of the contrast medium (black arrow). Embolization of the lesion **(E)**. Follow-up CT represented a marked decrease of the EDH **(F)**.

A patient in their 40s was admitted to the hospital after a fall and complained of a headache. The GCS score was 15. The CT scan identified a parietal fracture, a small acute EDH in the right parietal region adjacent to the fracture (Figures 2A,B). Ipsilateral ECA angiography revealed no significant abnormality (Figure 2C). However, super-selective MMA angiography showed abnormal dilation of branch vessels and extravasation of the contrast medium (Figure 2D). Embolization of the lesion was performed without complications (Figure 2E). The interval between the injury and the MMA embolization was <8 h. The post-operation CT scan demonstrated no evidence of hematoma enlargement, and the patient was discharged without any clinical deficits (Figure 2F).

### 4.3 Case 3

**Figure 3:**
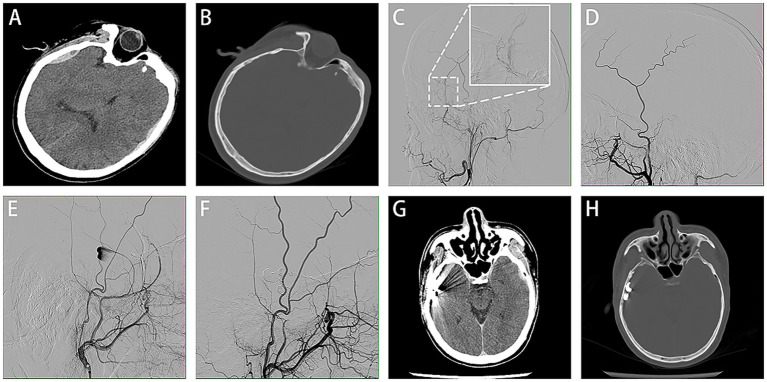
Case 3 representative images. Initial CT shows a left frontotemporal subdural hematoma and a right temporal EDH with an internal hypodense nodule **(A)** and an associated temporal bone fracture on bone window imaging **(B)**. Selective left ECA angiography revealed abnormal vascular dilatation with active contrast extravasation within the region outlined by the white dashed box. This area is shown at higher magnification in the white solid box **(C)**. The lesion was embolized subsequently **(D)**. Selective right ECA angiography showed a heart-shaped pseudoaneurysm originating from the posterior branch of the MMA **(E)**. The MMA was embolized promptly **(F)**. Follow-up CT confirmed that the size of the hematoma was not increased **(G,H)**.

An adult patient lost consciousness after crashing his electric bicycle and complained of headaches and nausea. The GCS score was 14/15. The CT scan revealed a left temporal cerebral contusion, a left frontotemporal subdural hematoma, and a right temporal EDH adjacent to a right temporal bone fracture. Additionally, a hypodense nodule was observed in the region of the EDH, which was suspected to be a vascular lesion (Figures 3A,B). Selective left ECA angiography revealed an abnormal expansion and active contrast extravasation, which was subsequently embolized (Figures 3C,D). Selective right ECA angiography showed a heart-shaped pseudoaneurysm in the posterior branch of the MMA (Figure 3E). The pseudoaneurysm ruptured during the embolization process; fortunately, prompt embolization of the MMA prevented further bleeding (Figure 3F), highlighting the fragility of traumatic pseudoaneurysms and the importance of prompt embolization once identified. After the operation, the CT scan showed no increase in bilateral hematoma, and the patient was released from the hospital without any neurological deficits (Figures 3G,H).

## 5 Discussion

EDH is a critical neurosurgical emergency that can be life-threatening due to its mass effect and requires emergency surgical evacuation. Nevertheless, small acute EDHs can be managed conservatively, and the optimal management remains undetermined to date. The current practice for the majority is meticulous neurological observation and repeated CT scans to monitor potential hematoma enlargement. Hematoma expansion occurs in 5.5 to 65% of cases, which probably leads to unpredictable sudden neurological deterioration (11, 12). Our data support a fracture-based risk stratification strategy in which early angiography and prophylactic MMA embolization are considered for patients whose fractures traverse the MMA course.

Unlike large epidural hematomas resulting from rupture of the proximal middle meningeal artery, small EDHs are more commonly attributable to injuries of distal MMA branches or post-traumatic MMA-diploic venous fistulas or pseudoaneurysms, particularly in the presence of skull fractures. These fracture-related vascular lesions may sustain persistent low-flow bleeding that is not readily detectable on initial CT imaging, thereby increasing the risk of delayed hematoma enlargement. From a clinical standpoint, skull fractures traversing the MMA groove or diploic space identify a subgroup of patients at heightened risk, in whom angiographic evaluation enables early detection of occult vascular pathology. In contrast, small EDHs without associated skull fractures are less likely to harbor ongoing arterial injury and can generally be managed conservatively with close clinical and radiological surveillance. This risk-stratified approach provides a rational framework for individualized management of small EDHs and supports selective angiographic assessment in patients most likely to benefit.

In recent years, MMA embolization has been successfully utilized for patients with small acute EDHs when hematoma evacuation is not required (13–16). Peres et al. (2) reported the results of a study involving 80 patients with small acute EDHs who received endovascular treatment, showing no rebleeding after embolization. This finding was compared to a literature-based control cohort comprising a total of 471 patients, 82 (17.4%) of whom were ultimately referred for surgical evacuation. Another study also demonstrated that 23% (37/160) of patients suffer EDH enlargement, leading to surgical evacuation during conservative management (17). Previous studies have shown that MMA embolization can significantly decrease the risk of hematoma expansion in small epidural hematomas. Our study further confirms this conclusion. Angiographic disappearance of extravasation indicates control of MMA branch bleeding and subsequent CT scans indicated no further hematoma expansion or hematoma regression. All patients were discharged without neurological deficits after MMA embolization.

Patients with skull fractures crossing over MMA are at increased risk of rebleeding and hematoma enlargement. Fractures can damage the MMA vessel walls, leading to unstable vascular injuries like pseudoaneurysms and dural fistula (6, 18). In our series, we observed that skull fractures accompanied all patients with small EDHs and could find vascular injuries in super-selective MMA angiography. Initially, we only concentrated on ECA angiography to detect vascular injuries. However, in one case, we struggled to find the vascular injury until we utilized a microcatheter to perform super-selective MMA angiography. This approach allowed us to successfully identify parallel and irregular linear roughened distal vascular wall of the MMA by MMA distal angiography, which we termed the “dual track sign”, which indicates vascular wall damage. Since then, we routinely performed super-selective MMA angiography in eligible patients to detect vascular injury. This allowed us to accurately identify injuries that might be overlooked by ECA angiography alone.

However, the optimal site for MMA embolization remains a matter of controversy. Some believe that the MMA should be embolized at the trunk of the MMA to save time and avoid unpredictable sudden rebleeding (19). In contrast, others argue that the microcatheter should be extended as far as possible to prevent the inappropriate embolism of dangerous anastomoses (2). For instance, the petrous branch of the MMA has anastomoses with the ascending pharyngeal artery. Furthermore, its sphenoid branch can enter the orbit to form an anastomosis with the ophthalmic artery. At our center, super-selective angiography of the MMA serves as an initial diagnostic step to identify potentially dangerous anastomoses and inform subsequent management decisions. If such connections are found, the microcatheter should be placed as far distally as possible to avoid the anastomosis; if there is no obvious dangerous anastomosis, embolization can be performed at the proximal end of the MMA main trunk.

Onyx^™^-18 is a nonadhesive liquid embolic agent, which is an ethylene vinyl alcohol co-polymer dissolved in DMSO with tantalum powder in suspension (20). The significant advantage of Onyx^™^-18 resides in its capacity to prevent adherence to the catheter and the vessel. This characteristic enables the operator to inject the substance slowly and accurately, facilitating real-time reflux monitoring and reducing the likelihood of catheter adhesion (21, 22). Meanwhile, coils are typically densely packed in the main trunks, which fail to reach small branches. NBCA polymerizes rapidly, which can limit injection time and distal penetration and may lead to incomplete occlusion in some settings. Additionally, Onyx is believed to provide more precise occlusion compared to NBCA (23). Therefore, we chose Onyx^™^-18 as the embolic agent in all cases. However, a drawback of Onyx embolization is that it must be dissolved in DMSO, which can cause intense pain in patients. At our center, before injecting the Onyx^™^-18, we usually administer a small amount (up to 50 mg) of 2% lidocaine into the MMA through the microcatheter to alleviate the intense pain caused by DMSO.

Patients with EDH enlargement often exhibit rapid clinical deterioration, necessitating urgent surgical evacuation. Current evidence highlights a critical temporal window for intervention, with approximately 80% of hematoma expansions requiring surgery occurring within 8 h post-injury (17), a phenomenon attributed to disrupted vascular integrity at the site of skull fractures. This aligns with the pathophysiological cascade triggered by traumatic MMA injury, where shearing forces from skull fractures induce pseudoaneurysm formation or active bleeding, exacerbating hematoma growth (6). Wang et al. further quantified this risk, reporting that 76% of progressive EDHs manifest within 6 h, while expansion beyond 24 h is exceptionally rare (<3%) (24). Such data underscore the imperative for early vascular assessment in high-risk populations. At our center, patients with small EDHs accompanied by skull fractures who are at high risk of hematoma expansion routinely undergo an emergency DSA to promptly assess any vascular injury, potentially obviating the need for surgical evacuation. Notably, all procedures performed under local anesthesia eliminate the need for general anesthesia, minimizing patient risks and costs, which are well tolerated and a critical advantage in emergency settings. The conventional approach focuses on careful monitoring. If the hematoma increases in size, a craniotomy will be required. Our research suggests that early intervention can prevent hematoma expansion. However, the available evidence remains observational and there are no comparative studies demonstrating a clear advantage of endovascular treatment over conservative management in this specific setting.

MMA pseudoaneurysms, though rare, may develop following trauma accompanied by a skull fracture (25). The fracture tears the vessel wall, but the tear is not large enough to result in an EDH, creating a clot that contains leaked blood. Then, the clot undergoes fibrous organization by surrounding tissues, and a pseudoaneurysm is formed. The pseudoaneurysm is more fragile and can be suddenly ruptured, leading to devastating consequences in 1–30 days after trauma, with peak vulnerability at 7–14 days (22, 26, 27). Gerosa et al. reported 11 cases of traumatic MMA pseudoaneurysms, 82% of which ruptured within 2 weeks if untreated (18). This delayed presentation poses diagnostic challenges, as patients may initially appear stable, but sudden pseudoaneurysm rupture leads to catastrophic EDH expansion, abrupt neurological decline, or even mortality. Consequently, to prevent progressive EDH, it is necessary to identify the vascular injury promptly. Among the 14 patients undergoing MMA embolization in our series, three cases (21.4%) were complicated by pseudoaneurysms, which demonstrated that pseudoaneurysms were not uncommon in patients with EDHs related to fractures. If these pseudoaneurysms are not treated promptly, they may rupture suddenly, resulting in disastrous consequences. In one case, a pseudoaneurysm ruptured intraprocedurally during catheter manipulation, likely due to mechanical stress or injection-related pressure changes on the fragile lesion. Prompt embolization of the MMA prevented further bleeding. Post-angiography confirmed complete occlusion of the rupture site, and follow-up CT scans demonstrated stable clot morphology without rebleeding. This case highlights the crucial role of proactive intervention in preventing delayed bleeding. These observations suggest a potential preventive role of early MMA embolization in a fracture-defined high-risk subgroup; however, prospective comparative studies are required to determine the clinical benefit and avoid overtreatment.

This study presents some limitations. First, as a retrospective single-center analysis, it is inherently subject to selection and information bias. Although we minimized confounding through strict inclusion criteria, the small sample size (*n* = 14) limits the statistical power to detect rare complications. Additionally, the predominance of patients with high GCS scores (78.6% with a GCS ≥ 13) and small hematoma volumes (mean 11.2 mL) may skew outcomes toward favorable prognoses, potentially underestimating the risks in severe TBI populations. To confirm our results, a randomized controlled trial (RCT) comparing early embolization versus conservative management in matched fracture-EDH cohorts is warranted. Second, the proactive embolization protocol initiated within 8 h post-injury raises questions about overtreatment. Approximately 30–50% of small EDHs remain stable without intervention, suggesting our 100% success rate in preventing expansion might reflect selection bias toward high-risk fractures rather than universal applicability. However, the consistent identification of MMA vascular lesions in all cases strongly supports the hypothesis that fracture-associated EDHs harbor vascular lesions requiring targeted management. Third, the study only includes patients with small EDHs and excludes those with EDHs who require surgical evacuation; we aim to explore the effects of MMA embolization before evacuation in the future. Finally, this proposed workflow just reflects our institutional experience in a selected high-risk subgroup and further multicenter studies are required before broader recommendations can be made.

## 6 Conclusion

Our findings suggest that early MMA embolization is a safe and effective strategy for preventing hematoma progression in high-risk small acute EDH patients with skull fractures. Routine vascular evaluation with super-selective DSA may be considered in selected patients within this high-risk population in our experience, as it may reduce monitoring burden and potentially lower the risk of delayed expansion.

## Data Availability

The raw data supporting the conclusions of this article will be made available by the authors, without undue reservation.
